# Supplementary material for: Untangling the seasonal dynamics of plant-pollinator communities
Source: Nat Commun. 2020 Aug 14;11:4086. doi: 10.1038/s41467-020-17894-y (PMC7429506; doi:10.1038/s41467-020-17894-y)
Supplement: Supplementary file 1 — Supplementary Information [file 41467_2020_17894_MOESM1_ESM.pdf]

Supplementary Information

Untangling the seasonal dynamics of plant-pollinator communities

Bramon Mora et al.

Supplementary Methods

Alignment matrix

We compiled all alignments between the networks in our dataset using the alignment matrix M. In particular, M contains information regarding every pairwise alignment between the networks $\{A, B, C, D...\}$ in our dataset—in our case, this corresponds to the pairwise alignment between 44 empirical networks. Given a set of n alignments $\{\lambda\}$ between A and B, we calculate a given element m_{ij}^{AB} as $\sum_{\lambda}^{n} k_{\lambda}$, where k_{λ} is 0 if species $i \in A$ and $j \in B$ are not paired in the alignment λ, and c_{ij} otherwise. Notice that c_{ij} characterizes the quality of the pairing i-j between $i \in A$ and $j \in B$ presented in the main text. The quality of the pairing i-j is calculated as the Pearson's correlation coefficient between the two species' motif-role profiles[1]. Specifically, this coefficient is equal to 1 if i and j have equivalent roles, 0 when there is no correlation between them, and -1 if they have opposite roles.

Relationship between group and position properties

To compare the different groups of positions, we studied the three position properties described in the main text using a multivariate autoregressive model. Specifically, we studied species' relative degree, neighbours' average relative degree and neighbours' maximum relative degree using species' position group and year of sampling. To do so, we used the R package *brms*[2] to fit a Bayesian multivariate linear regression. As outcome variables, we included, species' relative degree, neighbours' average relative degree, and neighbours' maximum relative degree. As categorical predictors, we included species' position group and year of sampling. We treated species' identity as a random effect, and added a first-order autoregression term using the week of sampling as a covariate and species' identity for every year as a grouping factor. To fit the Bayesian models, we chose weakly informative normally distributed priors—with mean $\mu = 0$ and standard deviation $\sigma = 1$—for the population-level effects; the default *brms* half Student-t priors with 3 degrees of freedom for the standard deviations of the group-level effects; and, the default *brms* flat priors bounded between -1 and 1 for the autocorrelation parameters. To calculate the effect sizes for the comparisons of the different predictions across position groups, we first drew 1000

samples from the different posterior distributions for a given position group and year using the 'pp_expect' function from the R package *brms*[2]. Then, we used the function 'cohen.d' of the R package *effsize*[3] to calculate the effect sizes for the comparison of every pair of posterior distributions.

Species structural dynamics across groups

As described in the main text, we examined the movement of pollinator and plant species across the different network positions using different probabilistic models. Given n groups of positions, our models describe a scenario in which every species can be found in $n+2$ possible states $\vec{y}$ at time t. These include: n states $y_1 \ldots y_n$ characterizing a species in each of the different groups of positions; a state $y_{n+1} = y_{\text{pre}}$ describing a species that has not yet entered the network; and a state $y_{n+2} = y_{\text{post}}$ describing a species that has already exited the network.

We estimated the rate of movement between the different states using Bayesian multinomial logistic regressions. In particular, our models considers $n+2$ types of events, describing the transition probability $\Pr\left(y_j | s_1\left(y_i, t\right), s_2\left(y_i, t\right), \ldots, s_{n+2}\left(y_i, t\right)\right)$ of species from any state y_i to any possible state y_j at time t following Eq. (1) from the main text. To infer the scores $s_k\left(y_i, t\right)$ that determine the resulting probabilities, we fixed one of them to serve as an arbitrary baseline—i.e. we assigned $s_1\left(y_i, t\right) = 0$—and estimated the remaining scores as $n+1$ linear models. The form of these linear models characterizes the type of transition probabilities considered. Specifically, we considered the following:

i) Constant transition probabilities. For this model, we defined the scores as

$$s_k\left(y_i\right) = \alpha_{k1} + \sum_{l=2}^{n+2} \alpha_{kl} \delta_{il}, \tag{1}$$

where α_{kl} are the parameters inferred by the model, and δ_{il} is a Kronecker delta that is set to 1 if $y_l = y_i$, and 0 otherwise. For any parameter α_{kl}, we chose weakly informative normally distributed priors, with mean $\mu = 0$ and standard deviation $\sigma = 10$.

ii) Constant transition probabilities accounting for sampling seasons as treat-

ments to the intercept. For this model, we defined the scores as

$$s_k\left(y_i, m\right) = \alpha_{k1} + \sum_{n=2}^{3} \gamma_k^n \delta_{mn} + \sum_{l=2}^{n+2} \alpha_{kl} \delta_{il}, \tag{2}$$

where $m = 1, 2, 3$ is a factor characterizing the sampling season; α_{kl} and γ_k^n are the parameters inferred by the model; and, δ_{mn} and δ_{il} are Kronecker deltas that are set to 1 if $m = n$ and $y_l = y_i$, respectively, and 0 otherwise. Notice that the parameters γ_k^n are used to treat the three sampling seasons as temporal replicates. For any parameter, we chose weakly informative normally distributed priors, with mean $\mu = 0$ and standard deviation $\sigma = 10$.

iii) **Constant probabilities with sampling seasons as group-specific treatments.** For this model, we defined the scores as

$$s_k\left(y_i, m\right) = \alpha_{k1} + \sum_{n=2}^{3} \gamma_{k1}^n \delta_{mn} + \sum_{l=2}^{n+2} \left(\alpha_{kl} + \sum_{n=2}^{3} \gamma_{kl}^n \delta_{mn} \right) \delta_{il}, \tag{3}$$

where $m = 1, 2, 3$ is a factor characterizing the sampling season; α_{kl} and γ_{kl}^n are the parameters inferred by the model; and, δ_{mn} and δ_{il} are Kronecker deltas that are set to 1 if $m = n$ and $y_l = y_i$, respectively, and 0 otherwise. Notice that the parameters γ_{kl}^n are used to treat the three sampling seasons as temporal replicates. For any parameter, we chose weakly informative normally distributed priors, with mean $\mu = 0$ and standard deviation $\sigma = 10$.

iv) **Time-dependent transition probabilities** This model is the one defined in the main text; therefore, scores are defined following Eq. (2) of the main text.

v) **Time-dependent transition probabilities accounting for sampling seasons as treatments to the intercept.** For this model, we defined the scores as

$$s_k\left(y_i, m, t\right) = \alpha_{k1} + \beta_{k1} t + \sum_{n=2}^{3} \gamma_k^n \delta_{mn} + \sum_{l=2}^{n+2} \left(\alpha_{kl} + \beta_{kl} t\right) \delta_{il}, \tag{4}$$

where $m = 1, 2, 3$ is a factor characterizing the sampling season; α_{kl}, β_{kl} and γ_k^n are the parameters inferred by the model; and, δ_{mn} and δ_{il} are Kronecker deltas that are set to 1 if $m = n$ and $y_l = y_i$, respectively, and 0 otherwise. Notice that the

parameters γ_k^n are used to treat the three sampling seasons as temporal replicates. For any parameter, we chose weakly informative normally distributed priors, with mean $\mu = 0$ and standard deviation $\sigma = 10$.

vi) Time-dependent probabilities with sampling seasons as group-specific treatments For this model, we defined the scores as

$$s_k(y_i, m, t) = \alpha_{k1} + \beta_{k1} t + \sum_{n=2}^{3} \gamma_{k1}^n \delta_{mn} + \sum_{l=2}^{n+2} \left(\alpha_{kl} + \beta_{kl} t + \sum_{n=2}^{3} \gamma_{kl}^n \delta_{mn} \right) \delta_{il}, \quad (5)$$

where $m = 1, 2, 3$ is a factor characterizing the sampling season; α_{kl}, β_{kl} and γ_{kl}^n are the parameters inferred by the model; and, δ_{mn} and δ_{il} are Kronecker deltas that are set to 1 if $m = n$ and $y_l = y_i$, respectively, and 0 otherwise. Notice that the parameters γ_{kl}^n are used to treat the three sampling seasons as temporal replicates. For any parameter, we chose weakly informative normally distributed priors, with mean $\mu = 0$ and standard deviation $\sigma = 10$.

Choosing the priors for the Bayesian models

As described in the main text, we defined the priors for the different parameters of the models as weakly informative normally distributed priors with mean $\mu = 0$ and standard deviation $\sigma = 10$. We did not have a preconceived notion of what these priors should be, and more restrictive priors–i.e. normally distributed with $\mu = 0$ and $\sigma = 1$—produced qualitatively the same results described in Fig. 4 of the main text. That said, we tested the design of model iv using simulated data, and these tests showed that less informative priors were needed in order to produce good estimates for some transition probability matrices.

Effect sizes for the comparison of the different transition probabilities

To understand the differences between the transition probabilities estimated by the Bayesian models, we compared the corresponding posteriors distributions using Cohen's *d*. Given any two transition probabilities described in the Results section of the main text at time t, we drew 1000 samples from their posterior distributions using the 'extract.samples' function of the R package *rethinking*[4]. Then, we used the function 'cohen.d' of the R package *effsize*[3]

to calculate the effect sizes for the comparison of these two posterior distributions.

Supplementary Notes

Supplementary Note 1: Relationship between position uniqueness and species richness

Bramon Mora et al.[1] showed that large food webs ($\gtrapprox$ 50 species) might be more redundant than smaller ones, as these have a greater probability of including trophically identical species as well as species that participate in just a single interaction. That is, species in larger networks might present lower position uniqueness than species in smaller networks. However, it is unclear to what extent uniqueness depends on species richness or is instead an intrinsic property of large ecological networks. For example, if a network of 10 species has a central node individually connected to the remaining 9 species, each of these 9 species will present very low position uniqueness. In contrast, if a network of 10 species is connected as two non-symmetric 5 species motifs, all 10 species will have high position uniqueness. This argument can be made for any number of species n. For the sake of this current study, nevertheless, all species show similar levels of position uniqueness, and if larger networks were significantly more redundant, we would expect to see lower levels of uniqueness in Fig. 2a from the main text. That is, controlling for species richness would not change the observed distribution in Fig. 2a in a way that would change our conclusions.

Supplementary Note 2: Relationship between species' relative degree and abundances

To study the link between species' relative degree and abundance, we used the abundance estimates from CaraDonna et al.[5]. For plant species, these were measured by independent counting of flowers and flower heads within a series of transects at the study site. On the other side, weekly pollinator abundances were estimated using the number of observation periods in which these were observed during the collection of plant-pollinator interaction data (see CaraDonna et al.[5] for details). Notice that since the abundance data for pollinator species was estimated from the interaction surveys from which their relative degree was calculated, one should expect the abundance measures to be at least somewhat dependent on the pollinators' observed number of interactions.

We studied the relationship between species' relative degree and abundance using two different models. First, and for the sake of simplicity, we used a linear regression to understand the overall trend of the relationship between these variables. We found a positive relationship between relative degree of plants and pollinators and their estimated abundance (Supplementary Fig. 9). The regressions presented R^2 values of 0.14 and 0.34 for plants and pollinators, respectively. Second, we used the Hill function to fit a more realistic scenario, where the relationship is nonlinear and absent species are expected to present 0 relative degree. In this case, we found the model to fit the data with a residual standard error of 0.2627 on 388 degrees of freedom for plants, and a residual standard error of 0.1928 on 850 degrees of freedom for pollinators (Supplementary Fig. 9). Overall, although we observed a positive relationship between relative degree and abundance of species, there is considerable variation (85.9% and 66.5% according to the linear regression for plants and pollinators, respectively) in species' relative degree that is not explained by abundance (Supplementary Fig. 9). Linear regressions including the year of sampling as an additional fixed effect support this observation, with R^2 values of 0.17 and 0.36 for plants and pollinators, respectively.

Supplementary Figures and Tables

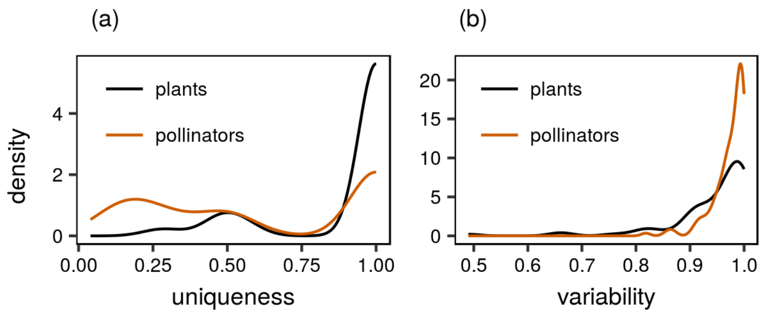

Supplementary Figure 1: Analysis of species' positions across binary networks. (a) Uniqueness of the position of plant and pollinator species within networks. (b) Variability of species' position across networks, including only plant and pollinator species that appear in multiple networks during a season.

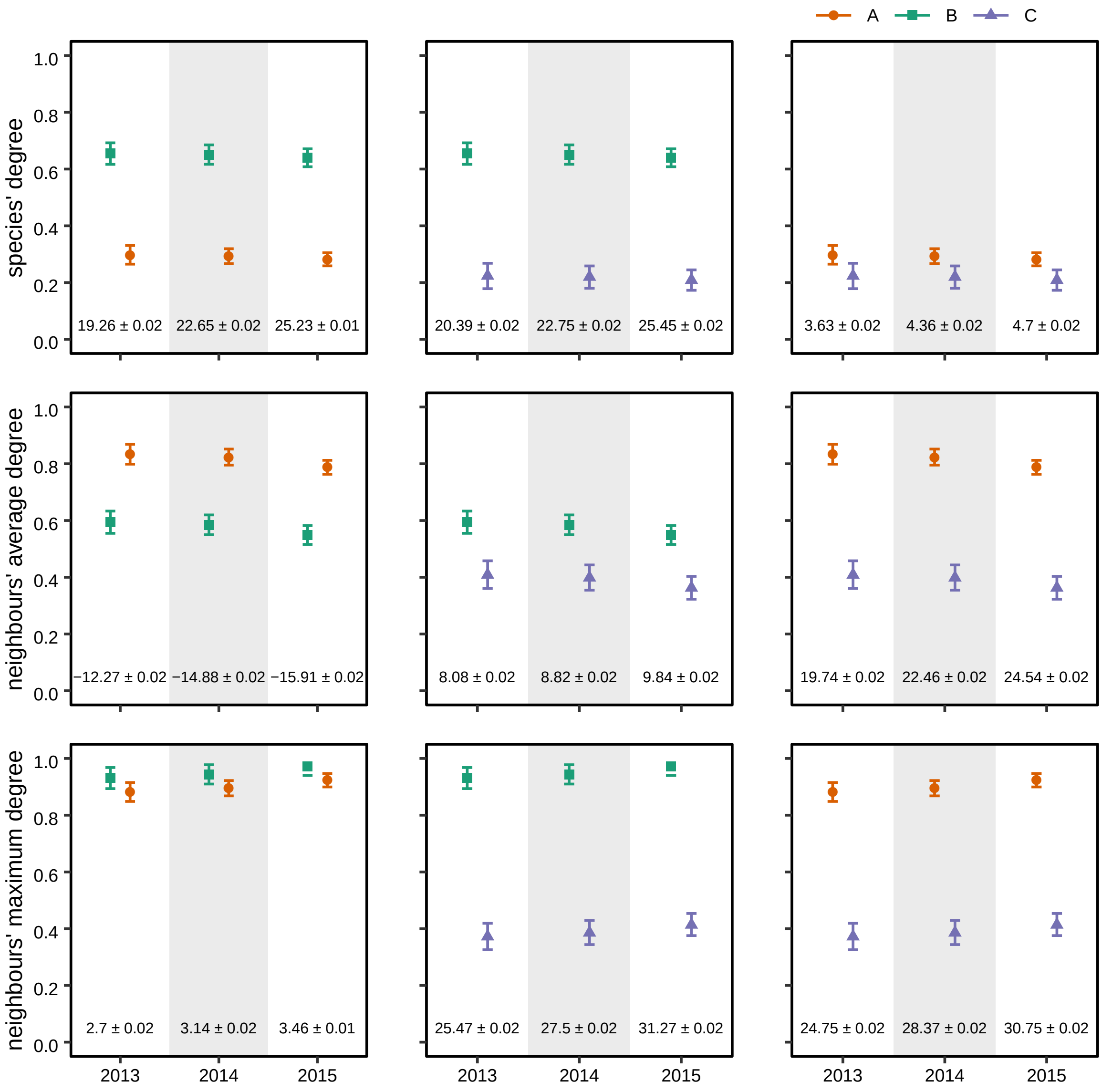

Supplementary Figure 2: Relationships between group and position properties. Every panel describes the comparison between the different properties studied (species' relative degree, neighbours' average relative degree and neighbours' maximum relative degree) for every pair of pollinator position groups (A, B, C). Each color and shape characterize the predictions of a different position group. The different columns of every panel describe the comparison of the predictions for every year of sampling. The points characterize the mean values, and the errorbars represent the credible intervals (2.5 and 97.5 percent quantiles).The scores along the x axis of every panel describe the mean effect size for each comparison (calculated as Cohen's d using 1000 samples from the different posterior distributions; see Supplementary Methods for further details). Just as an example, the panel in the top-left corner of the figure compares the degree of species from groups A and B, showing the predictions for every year of sampling.

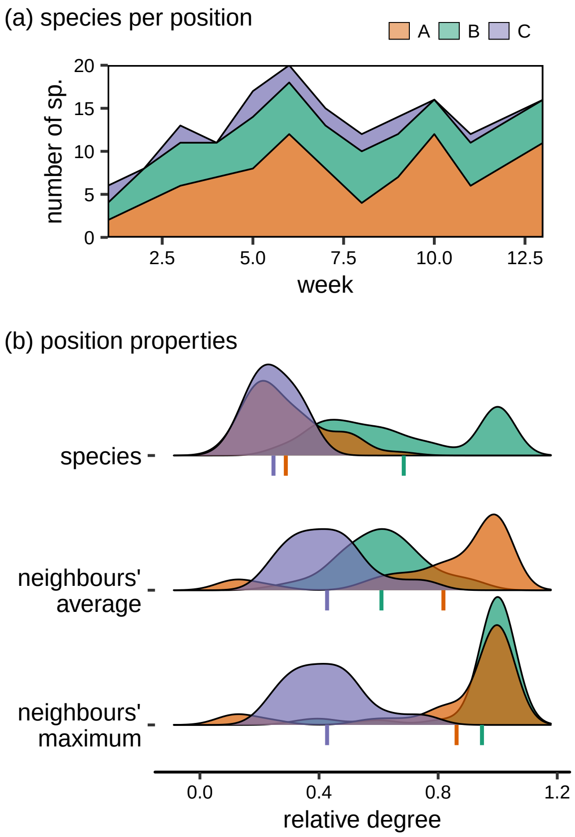

Supplementary Figure 3: Distinguishing properties of the different groups of pollinator positions found for the 2013 sampling season. (a) Number of pollinator species in each group of positions over time. Each color represents a different group of positions. (b) Different properties summarizing the species forming each group. The first panel depicts the relative degree distribution of the best connected neighbor of every species in each group. The second panel shows the neighbours' average relative degree for the species in each group. The third panel shows the relative degree distribution of species in each group. The colored segments depicted under the distributions characterize the mean of each distribution.

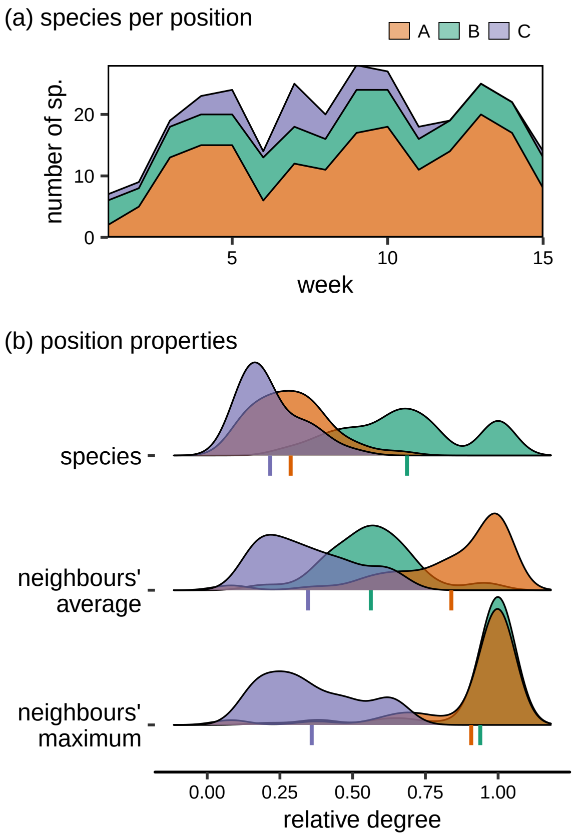

Supplementary Figure 4: Distinguishing properties of the different groups of pollinator positions found for the 2014 sampling season. (a) Number of pollinator species in each group of positions over time. Each color represents a different group of positions. (b) Different properties summarizing the species forming each group. The first panel depicts the relative degree distribution of the best connected neighbor of every species in each group. The second panel shows the neighbours' average relative degree for the species in each group. The third panel shows the relative degree distribution of species in each group. The colored segments depicted under the distributions characterize the mean of each distribution.

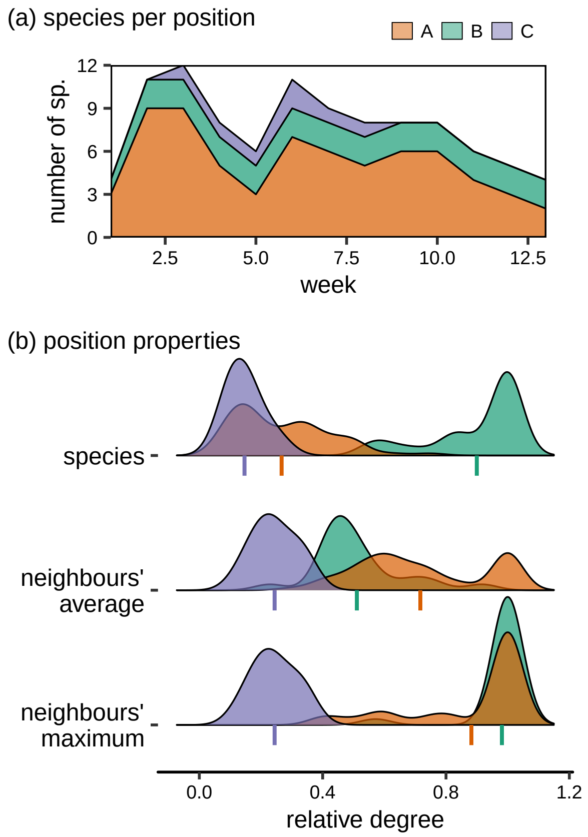

Supplementary Figure 5: Distinguishing properties of the different groups of plant positions found for the 2013 sampling season. (a) Number of plant species in each group of positions over time. Each color represents a different group of positions. (b) Different properties summarizing the species forming each group. The first panel depicts the relative degree distribution of the best connected neighbor of every species in each group. The second panel shows the neighbours' average relative degree for the species in each group. The third panel shows the relative degree distribution of species in each group. The colored segments depicted under the distributions characterize the mean of each distribution.

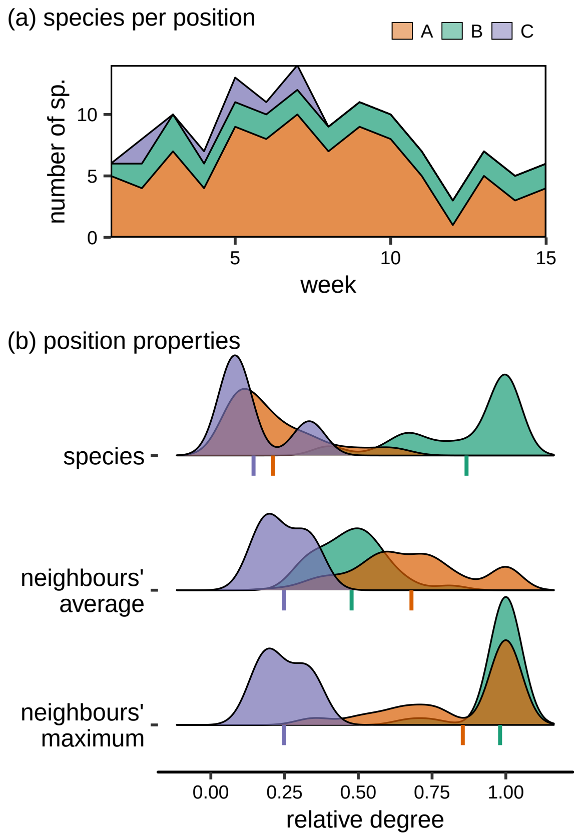

Supplementary Figure 6: Distinguishing properties of the different groups of plant positions found for the 2014 sampling season. (a) Number of plant species in each group of positions over time. Each color represents a different group of positions. (b) Different properties summarizing the species forming each group. The first panel depicts the relative degree distribution of the best connected neighbor of every species in each group. The second panel shows the neighbours' average relative degree for the species in each group. The third panel shows the relative degree distribution of species in each group. The colored segments depicted under the distributions characterize the mean of each distribution.

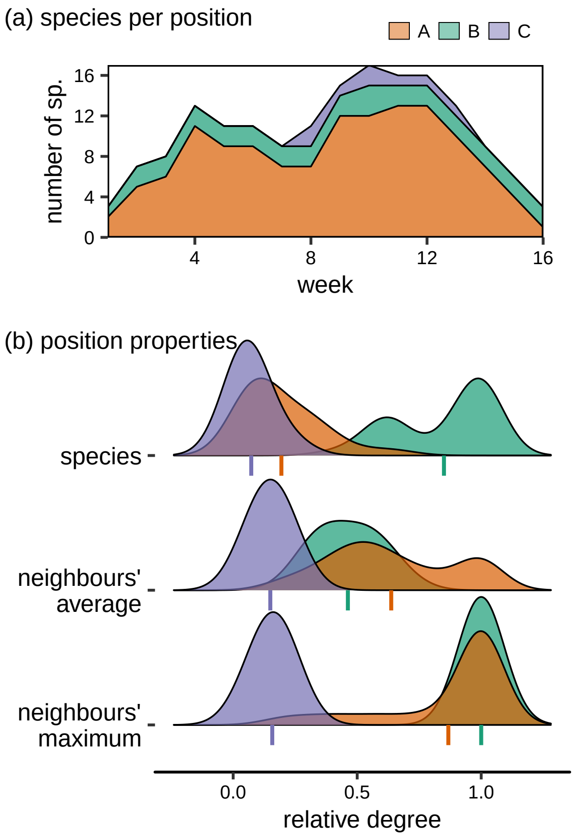

Supplementary Figure 7: Distinguishing properties of the different groups of plant positions found for the 2014 sampling season. (a) Number of plant species in each group of positions over time. Each color represents a different group of positions. (b) Different properties summarizing the species forming each group. The first panel depicts the relative degree distribution of the best connected neighbor of every species in each group. The second panel shows the neighbours' average relative degree for the species in each group. The third panel shows the relative degree distribution of species in each group. The colored segments depicted under the distributions characterize the mean of each distribution.

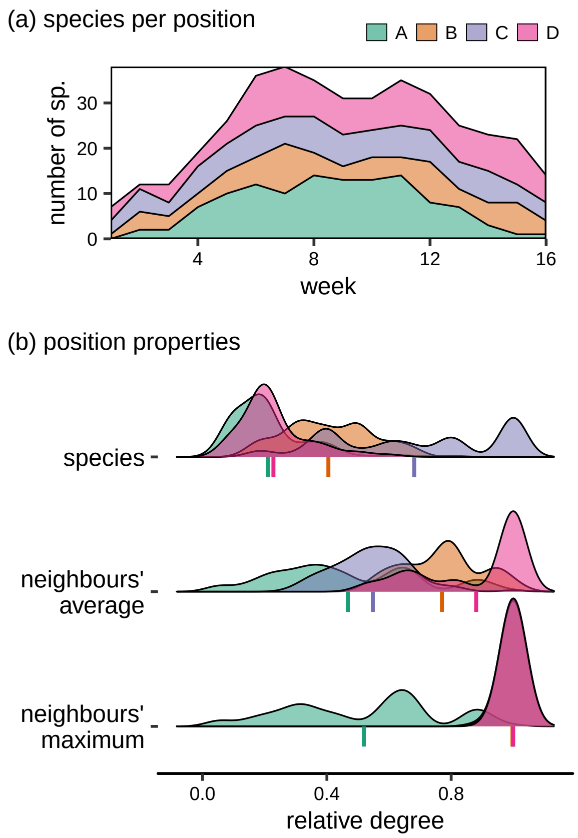

Supplementary Figure 8: Distinguishing properties of the different groups of species' position found for the 2015 sampling season using the multi-level community detection method (Table 1; Blondel et al.[6]). (a) Number of pollinator species in each group of positions over time. Each color represents a different group of positions. (b) Different properties summarizing the species forming each group. The first panel depicts the relative degree distribution of the best connected neighbor of every species in each group. The second panel shows the neighbours' average relative degree for the species in each group. The third panel shows the relative degree distribution of species in each group. The colored segments depicted under the distributions characterize the mean of each distribution.

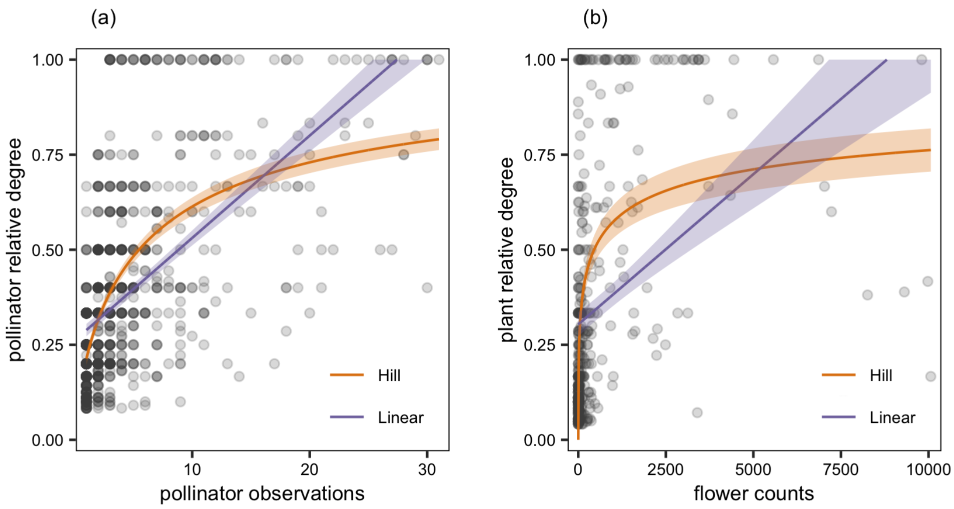

Supplementary Figure 9: Relationship between relative degree and abundance of plants and pollinators. The points represent the information of a plant or pollinator for a given week and year. The lines characterize the fit of a linear regression and the Hill function to the data. The shaded areas describe the confidence intervals around the fitted models. The estimated slopes for the linear regression $7.90 \times 10^{-5} \pm 9.90 \times 10^{-6}$ and $2.70 \times 10^{-2} \pm 0.13$ show a positive relationship between the relative degree and abundance of plants and pollinators, respectively. The estimated half-saturation density for the Hill function shows that the relationship vanishes at 453.01 ± 90.07 flower counts and 5.47 ± 0.25 pollinator observations.

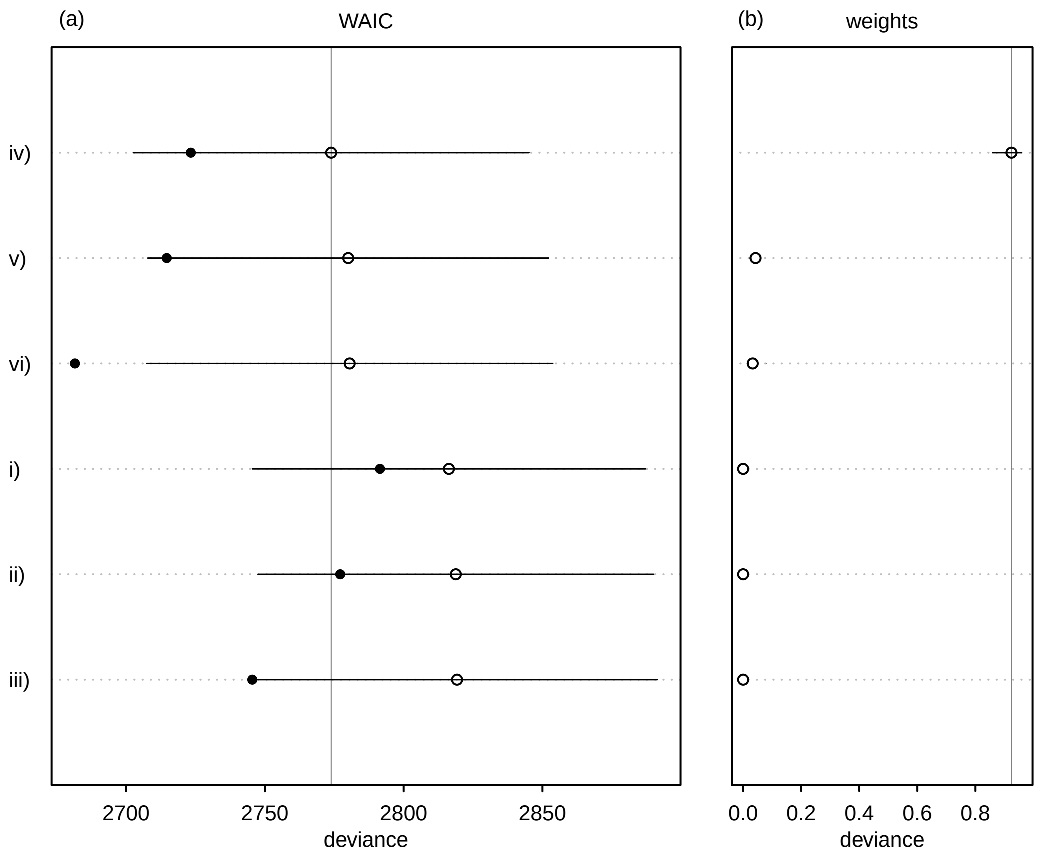

Supplementary Figure 10: Comparison of the different models using WAIC values. Each row describes one of the models from the 'Species dynamics across groups' section described above. Specifically, each row represents a model considering the following: i) constant transition probabilities; ii) constant transition probabilities accounting for sampling seasons as treatments to the intercept; iii) constant probabilities with sampling seasons as group-specific treatments; iv) time-dependent transition probabilities; v) time-dependent transition probabilities accounting for sampling seasons as treatments to the intercept; and vi) time-dependent probabilities with sampling seasons as group-specific treatments. (a) WAIC comparison of the different models. The filled points are the in-sample deviance of each model, and the open points are the estimated WAIC values. The dark line segments represent the standard error of each WAIC. The vertical line references the model with the lowest WAIC value. (b) Akaike weight for each model used to compare predictive accuracy of the different Bayesian models (see McElreath[4]). The open points are the mean Akaike weights and the line segments represent the corresponding standard error. The vertical line references the model with the highest Akaike weight. Note that the Akaike weights use the standard error of the difference between each WAIC and the best performing model to compare the relative predictive accuracy of the models. While the uncertainty in the WAIC values overlap between models, the measure shows that model iv) is the best model when accounting for the correlation between these uncertainties. The graphs were generated using some of the functions from the R package *rethinking*[4].

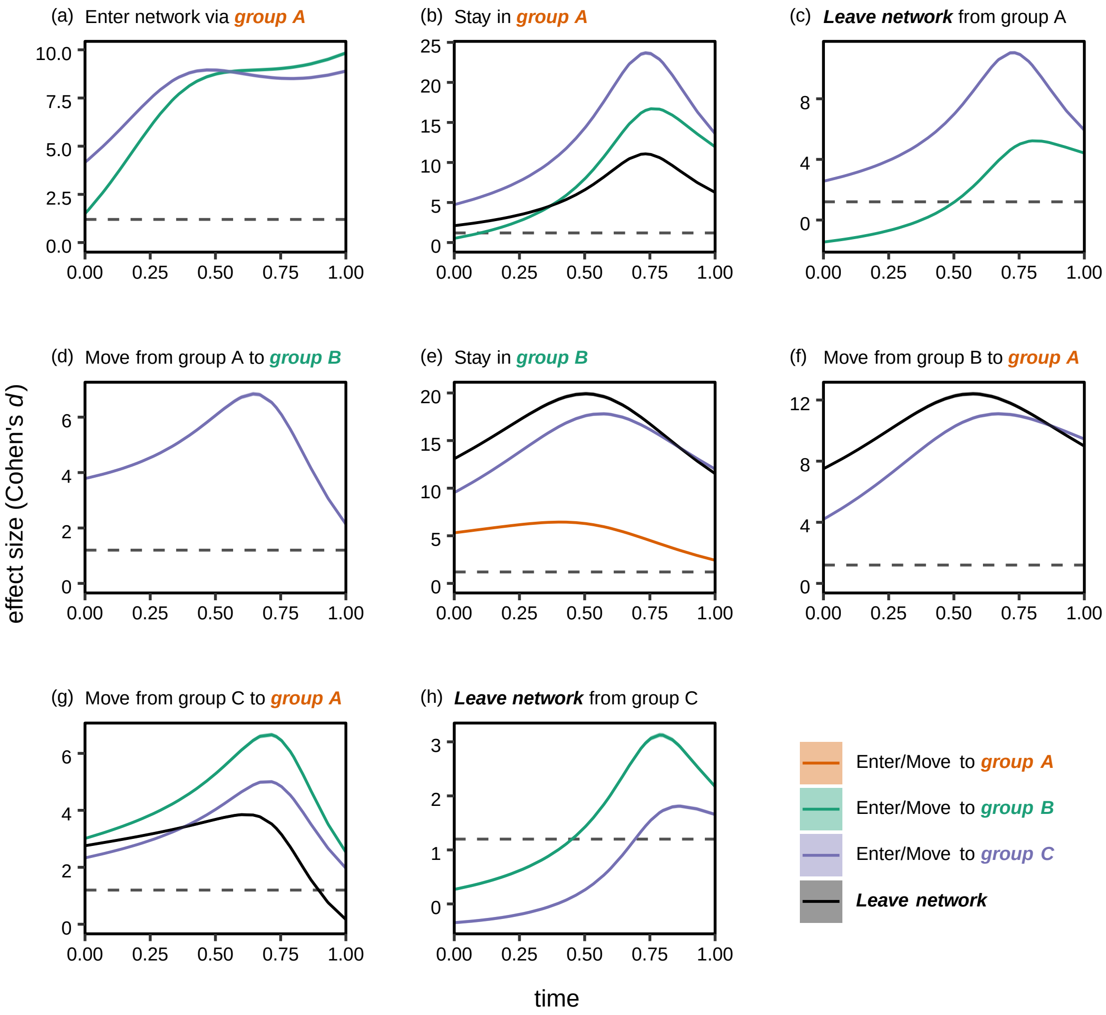

Supplementary Figure 11: Effect sizes for the conclusions reported in the "Results" section of main text. Every panel represents the effect sizes for the comparison between the time-dependent transition probabilities of Fig. 4. These are calculated based on differences between means, using Cohen's d (see Supplementary Methods for further details). Using the notation from Fig. 4, effect sizes are reported for the following comparisons: (a) 'enter in A' relative to 'enter in B' and 'enter in C'; (b) 'stay in A' relative to 'A→B', 'A→C' and 'exit from A'; (c) 'exit from A' relative to 'A→B' and 'A→C'; (d) 'A→B' relative to 'A→C'; (e) 'stay in B' relative to 'B→A', 'B→C' and 'exit from B'; (f) 'B→A' relative to 'B→C' and 'exit from B'; (g) 'C→A' relative to 'C→B', 'Stay in C' and 'exit from C'; and (h) 'exit from C' relative to 'C→B' and 'Stay in C'. The different lines characterize the estimated effect size, and the shade of each line characterizes the first and third quantiles. The dotted gray line at 1.20 indicates the threshold for which the effect sizes are considered to be very large[7].

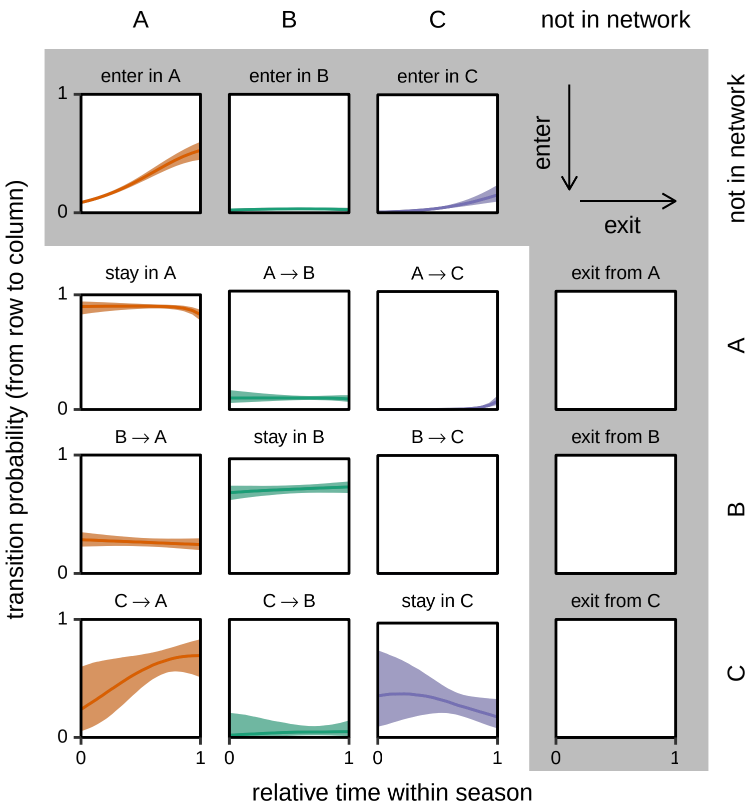

Supplementary Figure 12: Movement of plant species across position groups. Every panel in the matrix describes the transition probability between the different groups of positions (see model iv from Supplementary Methods). The order of the matrix is such that it characterizes the transition probabilities from row groups to column groups over time. The different groups are those presented in Supplementary Figs. 5–7. The shaded row describes the probabilities of species entering the network into the different groups (A, B and C) whereas the shaded column describes the probabilities of species exiting the network from each of these groups (the low values reflect the fact that plant species rarely leave the networks). The different lines in the graph represent the results found for every sampling year, where the shade of each line characterizes the first and third quantiles. The different lines in the graph represent the mean transition probabilities and the shade of each line characterizes the first and third quantiles. Notice that the color is chosen based on the recipient group of the transition probability.

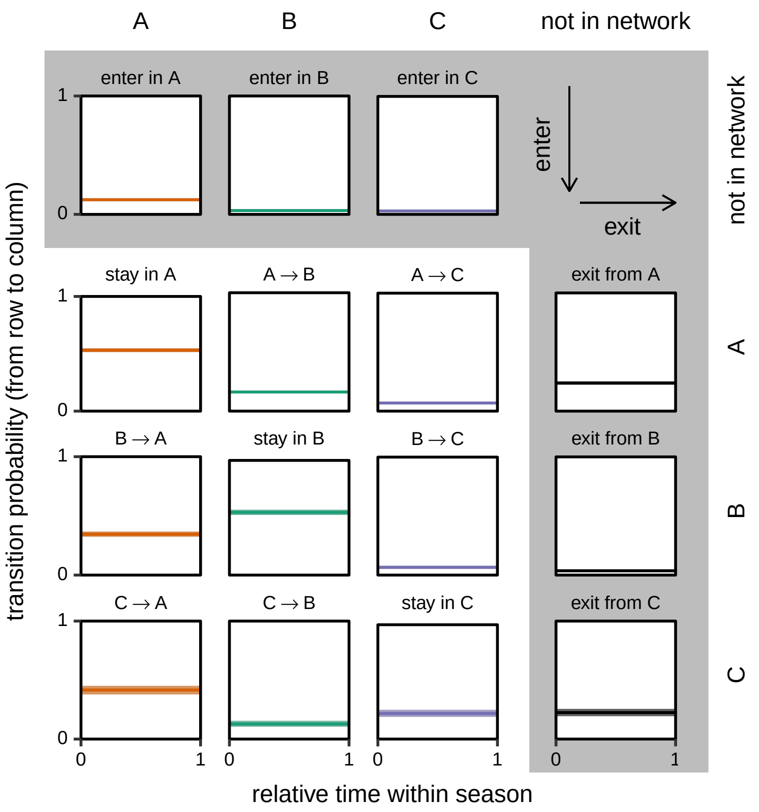

Supplementary Figure 13: Movement of pollinator species across position groups estimated using a model with constant transition probabilities (see model i from Supplementary Methods). Every panel in the matrix describes the transition probability between the different groups of positions. The order of the matrix is such that it characterizes the transition probabilities from row groups to column groups over time. The different groups are those presented in Fig. 3 of the main text. The shaded row describes the probabilities of species entering the network into the different groups (A, B and C) whereas the shaded column describes the probabilities of species exiting the network from each of these groups. The different lines in the graph represent the mean transition probabilities and the shade of each line characterizes the first and third quantiles. Notice that the color is chosen based on the recipient group of the transition probability.

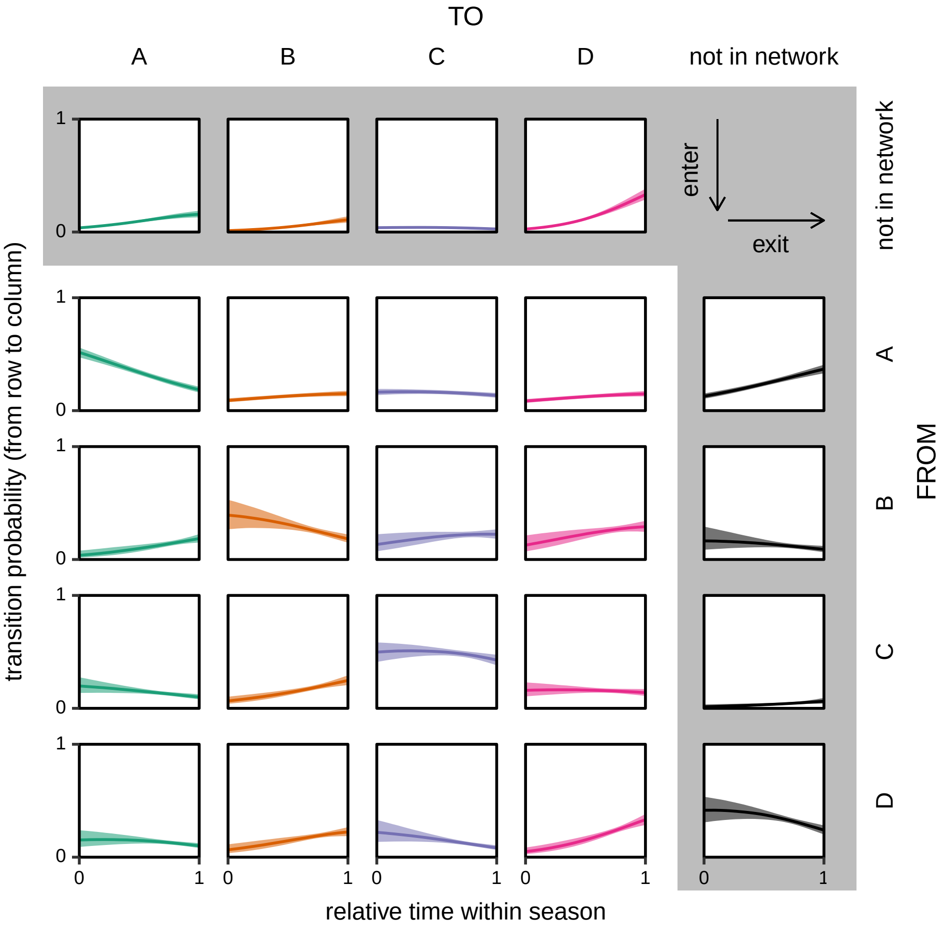

Supplementary Figure 14: Movement of pollinator species across position groups. Every panel in the matrix describes the transition probability between the different groups of positions (see model iv from Supplementary Methods). The order of the matrix is such that it characterizes the transition probabilities from row groups to column groups over time. The different groups are those presented in Supplementary Fig. 8. The shaded row describes the probabilities of species entering the network into the different groups (A, B, C and D) whereas the shaded column describes the probabilities of species exiting the network from each of these groups. The different lines in the graph represent the mean transition probabilities and the shade of each line characterizes the first and third quantiles. Notice that the color is chosen based on the recipient group of the transition probability.

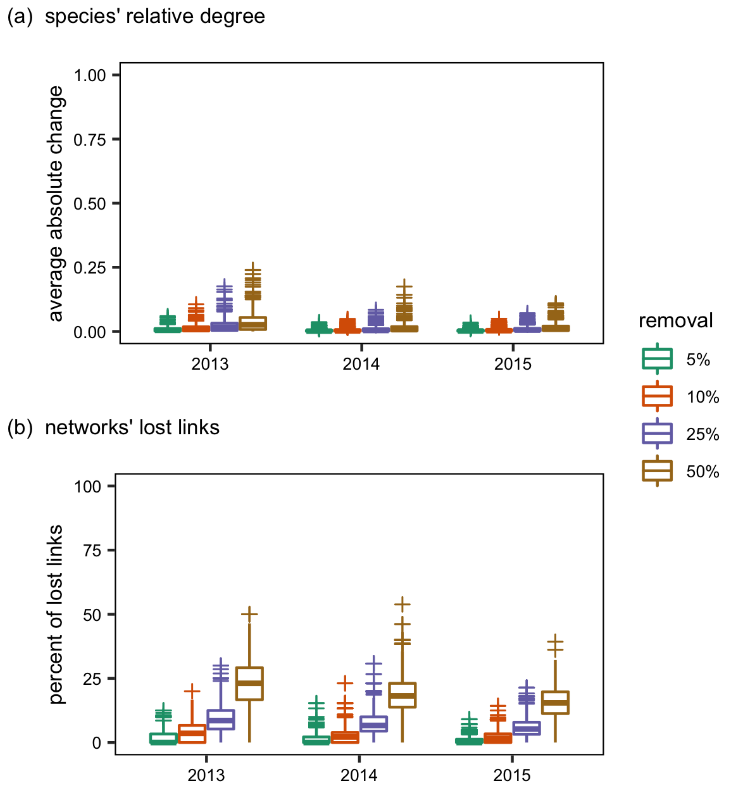

Supplementary Figure 15: Analysis of the sensitivity of species' relative degree and number of links to random removal of observations. (a) Sensitivity of species' relative degree to 5%, 10%, 25% and 50% random removal of observations for each different year. (b) Percent loss of links following the random removal of 5%, 10%, 25% and 50% of observations for each different year. The boxes in both panels characterize the median, and the the first and third quantiles; the line segments indicate the minima and maxima; and the whiskers represent the corresponding outliers.

	pol.	pl.	Normalized mutual information *greedy*	*betweenness*	*random walk*	*infomap*	*louvain*
greedy	2	2	1.00	0.69	0.66	0.59	0.67
betweenness	2	2	0.69	1.00	0.68	0.60	0.62
random walk	3	3	0.66	0.68	1.00	0.74	0.74
infomap	7	3	0.59	0.60	0.74	1.00	0.78
louvain	4	2	0.67	0.62	0.73	0.78	1.00

Supplementary Table 1: Community detection methods summary. The first column lists the community detection methods used here. The methods used are the following: greedy optimization (greedy[8]); edge betweenness (betweenness[9]); short random walks (random walks[10]); map equation optimization (infomap[11]); and, multi-level optimization (louvain[6]). The second and third columns describe the number of pollinator (pol.) and plant (pl.) groups found with each method. The rest of the columns provide a pairwise comparison of the results found with the different methods by means of the normalized mutual information criteria[12]. The methods highlighted are the ones used in the paper.

Supplementary References

[1] Bernat Bramon Mora, Dominique Gravel, Luis J Gilarranz, Timothée Poisot, and Daniel B Stouffer. Identifying a common backbone of interactions underlying food webs from different ecosystems. *Nat. Commun.*, 9(1):2603, 2018.

[2] Paul-Christian Bürkner et al. brms: An r package for bayesian multilevel models using stan. *J. Stat. Softw.*, 80(1):1–28, 2017.

[3] Marco Torchiano. effsize: efficient effect size computation. *R package version 0.8*, 1, 2017.

[4] Richard McElreath. *Statistical rethinking: A Bayesian course with examples in R and Stan.* Chapman and Hall/CRC, 2018.

[5] Paul J CaraDonna, William K Petry, Ross M Brennan, James L Cunningham, Judith L Bronstein, Nickolas M Waser, and Nathan J Sanders. Interaction rewiring and the rapid turnover of plant–pollinator networks. *Ecol. Lett.*, 20(3):385–394, 2017.

[6] Vincent D Blondel, Jean-Loup Guillaume, Renaud Lambiotte, and Etienne Lefebvre. Fast unfolding of communities in large networks. *J. Stat. Mech.: Theory E*, 2008(10): P10008, 2008.

[7] Shlomo S Sawilowsky. New effect size rules of thumb. *J. Mod. Appl. Stat. Methods*, 8 (2):597–599, 2009.

[8] Aaron Clauset, Mark EJ Newman, and Cristopher Moore. Finding community structure in very large networks. *Phys. Rev. E*, 70(6):066111, 2004.

[9] Mark EJ Newman and Michelle Girvan. Finding and evaluating community structure in networks. *Phys. Rev. E*, 69(2):026113, 2004.

[10] Pascal Pons and Matthieu Latapy. Computing communities in large networks using random walks. *J. Graph Algorithms Appl.*, 10(2):191–218, 2006.

[11] Martin Rosvall, Daniel Axelsson, and Carl T Bergstrom. The map equation. *Eur. Phys. J. ST*, 178(1):13–23, 2009.

[12] Leon Danon, Albert Diaz-Guilera, Jordi Duch, and Alex Arenas. Comparing community structure identification. *J. Stat. Mech.: Theory E*, 2005(09):P09008, 2005.
