# Supplementary material for: Untangling the seasonal dynamics of plant-pollinator communities
Source: Nat Commun. 2020 Aug 14;11:4086. doi: 10.1038/s41467-020-17894-y (PMC7429506; doi:10.1038/s41467-020-17894-y)
Supplement: Supplementary file 3 — Reporting Summary [file 41467_2020_17894_MOESM3_ESM.pdf]

nature research

Corresponding author(s): Bernat Bramon Mora

Last updated by author(s): Jul 22, 2020

Reporting Summary

Nature Research wishes to improve the reproducibility of the work that we publish. This form provides structure for consistency and transparency in reporting. For further information on Nature Research policies, seeAuthors & Referees and theEditorial Policy Checklist .

Statistics

For all statistical analyses, confirm that the following items are present in the figure legend, table legend, main text, or Methods section.

n/a	Confirmed	
☐	☒	The exact sample size (*n*) for each experimental group/condition, given as a discrete number and unit of measurement
☐	☒	A statement on whether measurements were taken from distinct samples or whether the same sample was measured repeatedly
☒	☐	The statistical test(s) used AND whether they are one- or two-sided<br>*Only common tests should be described solely by name; describe more complex techniques in the Methods section.*
☐	☒	A description of all covariates tested
☐	☒	A description of any assumptions or corrections, such as tests of normality and adjustment for multiple comparisons
☐	☒	A full description of the statistical parameters including central tendency (e.g. means) or other basic estimates (e.g. regression coefficient) AND variation (e.g. standard deviation) or associated estimates of uncertainty (e.g. confidence intervals)
☒	☐	For null hypothesis testing, the test statistic (e.g. *F*, *t*, *r*) with confidence intervals, effect sizes, degrees of freedom and *P* value noted<br>*Give P values as exact values whenever suitable.*
☐	☒	For Bayesian analysis, information on the choice of priors and Markov chain Monte Carlo settings
☐	☒	For hierarchical and complex designs, identification of the appropriate level for tests and full reporting of outcomes
☐	☒	Estimates of effect sizes (e.g. Cohen's *d*, Pearson's *r*), indicating how they were calculated

Our web collection on statistics for biologists contains articles on many of the points above.

Software and code

Policy information about availability of computer code

Data collection	No software was used for data collection
Data analysis	Code to conduct the network alignment described will be made available upon request. To calculate the motif-role profiles, we used the pymfinder software that is currently available as a preprint in https://doi.org/10.1101/364703. To generate the posterior samples for the Bayesian models we used the R packages 'rstan' (version 2.19.1) and 'brms' (version 2.13.3). To calculate the effect sizes for the comparison between posterior distributions, we used the R package 'effsize' (version 0.8). To generate supplementary figure 10, we used the R package 'rethinking' (version 2.11).

For manuscripts utilizing custom algorithms or software that are central to the research but not yet described in published literature, software must be made available to editors/reviewers. We strongly encourage code deposition in a community repository (e.g. GitHub). See the Nature Research guidelines for submitting code & software for further information.

Data

Policy information about availability of data

All manuscripts must include a data availability statement. This statement should provide the following information, where applicable:

- Accession codes, unique identifiers, or web links for publicly available datasets
- A list of figures that have associated raw data
- A description of any restrictions on data availability

The primary data associated with this manuscript are available in the Environmental Data Initiative (EDI) digital repository: https://doi.org/10.6073/pasta/27dc02fe1655e3896f20326fed5cb95f

Field-specific reporting

Please select the one below that is the best fit for your research. If you are not sure, read the appropriate sections before making your selection.

- [ ] Life sciences
- [ ] Behavioural & social sciences
- [x] Ecological, evolutionary & environmental sciences

For a reference copy of the document with all sections, see nature.com/documents/nr-reporting-summary-flat.pdf

Ecological, evolutionary & environmental sciences study design

All studies must disclose on these points even when the disclosure is negative.

Study description	We studied the seasonal dynamics of plant-pollinator communities by analysing network time series (presented by Caradonna et. al., Ecology Letters 2019)
Research sample	We studied plant-pollinator interaction networks from a subalpine community in the Colorado Rocky Mountains (Caradonna et al., Ecology Letters 2019). These data were sampled by Caradonna et al. at weekly intervals over three summer growing seasons, and contain nearly 30000 pairwise interactions between a total of 93 pollinator species and 46 flowering plants. To study the dynamics of these plant-pollinator communities, we aggregated the observed interactions into weekly plant-pollinator networks, where the weight of all interactions was set to the absolute number of observed interactions between the corresponding species pair during that week. In total, this resulted in three seasonal network time series comprising 12, 15, and 16 weekly weighted networks, respectively (see Caradonna et. al., Ecology Letters 2019 for further details).
Sampling strategy	We chose to use the data presented by Caradonna et. al. because this describes well resolved weekly plant-pollinator networks (based on sensibility analyses by Caradonna et al. showing consistent sampling and average detection of interactions for the networks). Moreover, this dataset contains three years that could be used as replicates. To our knowledge, this was the best resolved dataset for our study and questions answered in the manuscript.
Data collection	We did NOT collect experimental data. In Caradonna et. al. (Ecology Letters 2019), one can read: "Within each week, we conducted 32 15-min observation periods for a total of 8 h per week. Each complete weekly interaction census (i.e. all 32 observation periods) took place over 2–3 consecutive days and was separated from the start of the next weekly census by 3–5 days. We randomly selected one of four quadrants within each meadow during each 15-min observation period, we then sampled the remaining quadrants in random order, and then repeated this in the other meadow; we alternated the starting meadow on successive days. During each 15-min observation period, we walked around the focal quadrant and recorded all observed plant–pollinator interactions. We defined an interaction as taking place when a floral visitor of any species unambiguously contacted the reproductive structures of flowers; we refer to floral visitors as pollinators while recognising that their quality as mutualists may vary widely. To prevent observer bias, observations in each season were made by the same two researchers (PJC and JLC in 2013, PJC and RMB in 2014 and 2015). All observations took place between 0900 and 1700 h during weather conditions favourable for pollinator activity (ambient temperature > 8°C, not snowing or raining)."
Timing and spatial scale	We did NOT collect experimental data. With that being said, according to Caradonna et. al. 2019, observations began about 1 week after snowmelt each year (coinciding with the first emergence of flowers and pollinators). The data collection was done during the flowering seasons (11 weeks in 2013, 15 weeks in 2014 and 16 weeks in 2015). With regard to the spatial scale of the study, according to Caradonna et al., all observations took place in two adjacent dry meadows that cover c. 2800 and 3015 m^2, respectively, and are separated by c. 100 m of forest.
Data exclusions	We did not exclude any of the experimental data collected by Caradonna et. al. 2019.
Reproducibility	We did NOT collect experimental data. The dataset contains three replicates corresponding to the three sampling seasons. Our work analyzed this data using multiple models that considered these replicates together and separately, finding consistent results for each replicate.
Randomization	We did NOT collect experimental data, and this section is therefore not relevant for our study.
Blinding	All analyses were blind as there was no expected answer.

Did the study involve field work? [ ] Yes [x] No

Reporting for specific materials, systems and methods

We require information from authors about some types of materials, experimental systems and methods used in many studies. Here, indicate whether each material, system or method listed is relevant to your study. If you are not sure if a list item applies to your research, read the appropriate section before selecting a response.

Materials & experimental systems

n/a	Involved in the study
☒	☐ Antibodies
☒	☐ Eukaryotic cell lines
☒	☐ Palaeontology
☒	☐ Animals and other organisms
☒	☐ Human research participants
☒	☐ Clinical data

Methods

n/a	Involved in the study
☒	☐ ChIP-seq
☒	☐ Flow cytometry
☒	☐ MRI-based neuroimaging
